# Approaches and Challenges in Managing Treatment-Resistant Hypertension: A Case Report

**DOI:** 10.7759/cureus.75554

**Published:** 2024-12-11

**Authors:** Julian Morano, Samer Kholoki

**Affiliations:** 1 Internal Medicine, Saint James School of Medicine, The Valley, AIA; 2 Internal Medicine, University of Chicago Medicine AdventHealth La Grange, Chicago, USA

**Keywords:** blood pressure management, medical comorbidities, polypharmacy, renal fibromuscular dysplasia, treatment-resistant hypertension

## Abstract

Treatment-resistant hypertension (TRH) is defined by consistently elevated blood pressure readings unresponsive to medical management. In clinical practice, it poses a significant challenge due to the intertwining variables that may cause the issue to persist such as lifestyle, genetics, and other comorbidities, as opposed to simple medication non-adherence. This report describes the case of a 68-year-old female patient presenting for a routine follow-up with persistently elevated ambulatory blood pressure readings. The patient was compliant with her medication regimen and followed lifestyle modification guidelines. Physical exam and review of systems showed no changes relative to previous visits. Laboratory findings were insignificant other than a low estimated glomerular filtration rate (eGFr) of 52. The patient's condition was stable but required further management to bring blood pressure readings into or closer to optimal range. This case looks at how the patient was managed and the rationale behind these decisions based on the patient's clinical history. It also emphasizes the importance of regular monitoring and constant adjustment of medical management to treatment for patients with TRH due to its unrelenting nature.

## Introduction

Treatment-resistant hypertension (TRH) is characterized by a persistently elevated blood pressure greater than 140/90, unresponsive to medical management. The diagnosis is made when three antihypertensive agents, prescribed in combination, fail to adequately reduce blood pressure to within normal limits, or when four antihypertensive agents, prescribed in combination, are required to reach adequate blood pressure levels. The etiology of TRH is not straightforward, requiring careful consideration of the patient's comorbidities, lifestyle, and genetic differences. The implications of a better understanding of how to treat and manage TRH are immense, where chronic uncontrolled high blood pressure has gained its reputation as the “silent killer” through multisystemic end organ damage such as strokes, cardiovascular disease, and kidney failure. 

The increasing prevalence of TRH has caused it to gather more clinical attention. One study indicated that as many as 100-500 million individuals may be affected [1] while a meta-analysis of 3.2 million patients states that the overall prevalence of TRH was about 10% of all hypertensive patients [2]. Moreover, a study of 13,375 hypertensive individuals showed that TRH has increased from 15.9% (1998-2004) to 28.0% (2005-2008) in individuals being treated for hypertension [3]. Further, it has been found that TRH is more commonly found in those with high BMI, coronary artery disease, cerebrovascular accidents and transient ischemic attacks, peripheral vascular disease, congestive heart failure, chronic kidney disease (CKD), diabetes mellitus, and metabolic syndrome [4]. As per Boersma et al., the prevalence of comorbidities among the adult population in the United States is increasing and their contribution to the overall issue of TRH is inevitable [5]. 

Pharmacologic therapy is necessary to target the major physiological pathways causing TRH. Polypharmacy is almost guaranteed where typical management includes a diuretic, angiotensin-converting enzyme inhibitor (ACEi) or angiotensin receptor blocker (ARB), calcium channel blocker (CCB), and β-blocker. Each drug may be titrated to its maximum recommended dose in addition to medications from other classes unless there are adverse side effects. Alternative medications may be used based on the patient's clinical history and are an important part of creating an individualized treatment plan. Medications like spironolactone, eplerenone, or aliskiren may be prioritized for patients with hyperaldosteronism whereas β-blockers may be prioritized in those with coronary artery disease or heart failure. In unresponsive cases, alpha-agonists or vasodilators may be used [6]. Alongside pharmacotherapy, lifestyle modification is also required to achieve maximum therapeutic effect. In a study of 140 individuals with a structured diet and exercise program, significant reductions in ambulatory blood pressure were seen [7]. 

This report aims to highlight the challenge of treating TRH in patients who have multiple other comorbidities. TRH requires a nuanced, individualized approach to treatment, and this report seeks to offer valuable insights for healthcare providers in optimizing management strategies. By contributing to the growing body of data on TRH, the case underscores the importance of research aimed at refining clinical protocols and enhancing resource allocation for more effective patient care. It also emphasizes the critical need for personalized therapeutic interventions to improve patient outcomes and better address the challenges of managing TRH in the presence of concurrent health issues. 

## Case presentation

A 68-year-old female patient with a complex medical history, including atrial fibrillation, bilateral renal artery stenosis, fibromuscular dysplasia, abdominal aortic aneurysm, hyperlipidemia, hypertension, and mild tricuspid and aortic regurgitation, presented for a follow-up appointment at her primary care physician's office to re-evaluate her TRA. Her past surgical history included coronary artery bypass grafting (CABG) in her mid-30s, bilateral carotid endarterectomies, and iliac stent placement. The patient had initially developed hypertension in her early 60s, and physical examination revealed bilateral bruits. The patient was later found to have bilateral carotid artery occlusions due to fibromuscular dysplasia, causing her to have headaches and episodic hypertension due to impaired cerebral perfusion. At this time the patient was already on carvedilol and clonidine, and nifedipine was also initiated to help reduce carotid vasoconstriction. Five years prior to the current presentation, she underwent bilateral carotid endarterectomies to address the occlusions, and nifedipine was discontinued. A year ago, the patient presented for follow-up with persistently elevated blood pressure.

The patient stated she measured her blood pressure once or twice daily and noticed that her systolic pressure had been consistently in the 140-170 mmHg range, often being closer to the latter. She had a BMI of 26.2 kg/m^2^. The patient did not smoke and had one to two glasses of wine per week. Physical exam showed the absence of carotid bruits but faint abdominal bruits and no other physical symptoms. The patient also had labs drawn and pertinent values included an estimated glomerular filtration rate (eGFr) of 56, creatinine of 1.2, potassium of 3.6, and blood urea nitrogen (BUN)/creatinine ratio of 18:1. Given the patient's history of fibromuscular dysplasia causing vascular constrictions and declining eGFR along with signs of decreased blood volume reaching the kidney, there was high suspicion for the development renal artery stenosis which was later diagnosed. Despite a medication regimen including metoprolol, atorvastatin, aspirin, and clopidogrel, her blood pressure remained poorly controlled. A decision was made to initiate torsemide and nifedipine; however, nifedipine was again discontinued due to intolerable side effects, including pruritus and bruising. It was subsequently replaced with olmesartan.

The patient was advised to monitor ambulatory blood pressure twice daily, remain compliant with medications, and return every one to two months for follow-up visits. At each visit, blood pressure logs would be reviewed, the patient would be examined for signs of end organ damage, and medication reconciliation would be performed with adjustments made based on blood pressure values and patient feedback. Labs would be drawn twice per year to monitor kidney function and other parameters. The patient was also advised to return to the clinic if there were any acute changes or go to the emergency department if there were any red-flag symptoms. 

Since then, the patient has presented routinely to monitor blood pressure readings and adjust her medication doses. At her most recent visit, laboratory findings were largely unremarkable compared to previous visits, with the exception of a mildly reduced estimated glomerular filtration rate (eGFR 52). Despite adherence to an extensive pharmacological regimen, including olmesartan, carvedilol, torsemide, clonidine, clopidogrel, rosuvastatin, ezetimibe, aspirin, celecoxib, and acetaminophen, the patient's blood pressure remained sub-optimally controlled, necessitating ongoing management adjustments.

## Discussion

TRH continues to be a clinically significant challenge, especially in patients who have extensive medical histories. The current case highlights how implementing pharmacotherapy and interrupting the physiologic mechanisms that contribute to TRH can be insufficient even when following current guidelines. 

FMD is a noninflammatory vascular disease that primarily affects women aged 20-60 years. It may affect any artery in the body and can have symptoms ranging from stroke and coronary artery disease to hypertension [8]. The presenting patient developed bilateral carotid artery occlusions and spiking blood pressure, but this was resolved with bilateral carotid endarterectomies. It must be noted the disease is likely participating in other vascular abnormalities seen in the patient such as her abdominal aortic aneurism, early CABG, and later renal artery stenosis. The increasingly poor perfusion to the kidneys resulted in the activation of the renin-angiotensin-aldosterone system (RAAS) and subsequent sodium retention; both major contributors to increasing blood pressure [9]. In relation to renal artery stenosis, the patient likely developed CKD as a result, with an eGFR of 52. As mentioned by Sarafidis et al., treatment resistance often develops due to the RAAS system activation and impaired excretion of sodium as a result of CKD [10]. The patient's history of a CABG in her mid-30s also raises concerns for higher-than-normal atherosclerotic plaque buildup and increased arterial stiffness given her age and history of FMD. Although the patient's lipids were within normal range, arterial stiffness is a physiological change that occurs with natural aging regardless of other pathologies [11]. Further, the patient's history of aortic regurgitation, although mild, can also contribute to hypertension. When there is retrograde flow in the cardiac cycle, the heart must pump more blood in order to maintain a constant cardiac output, thus increasing systolic blood pressure while reducing diastolic pressure. This is not always present in cases of aortic regurgitation but allows one to see how comorbidities can compound and affect blood pressure [12]. 

The patient's complex medication regimen of 10 mg olmesartan daily, 6.25 mg carvedilol daily, 10 mg torsemide daily, 0.2 mg clonidine daily, and other medications is an attempt at reducing the patient's blood pressure while treating other comorbid conditions. The typical protocol for TRH is an ACE/ARB, β-blocker, diuretic, and CCB. Currently, the patient is on three of the medications listed above as well as an alpha-2 adrenergic agonist, clonidine. The patient has been titrated to maximal doses or maximally tolerated doses of each medication. For example, torsemide dosage has been maintained at 10 mg daily due to urinary frequency, carvedilol has been maintained at 6.25 mg daily to prevent bradycardia, and olmesartan is being used cautiously to prevent renal deterioration. The rationale behind the use of these medications lies in the standard management of hypertension and the associated comorbidities seen in the patient. Olmesartan is an ARB that interrupts the RAAS system and angiotensin 2 (AT2) and prevents the development of hypertension through different mechanisms. First, it inhibits the direct vasoconstrictive effect of AT2 directly reducing blood pressure. Next, it inhibits anti-diuretic hormone release preventing volume expansion. Finally, it inhibits the release of aldosterone which allows for sodium and water reabsorption [13]. This is valuable in reducing the patient's hypertension but must be used carefully as it can worsen the patient's CKD and further complicate treatment. Torsemide contributes by reducing overall blood volume, and carvedilol is an effective α- and β-blocker reducing sympathetic transmission. This is necessary to prevent vascular constriction while reducing cardiac oxygen demand in a patient with a history of CAD and atrial fibrillation. While not a part of typical therapy, clonidine can be used during higher-than-normal elevations in the patient's blood pressure by reducing adrenergic outflow [14]. Other pertinent medications include clopidogrel, aspirin, rosuvastatin, ezetimibe. These medications play an important role in preventing arterial thrombus formation given a history of CAD, but they also have secondary implications. Statins such as rosuvastatin have been found to have protective effects and reduce arterial wall stiffness, not only by lowering low-density lipoprotein (LDL) but also through its anti-inflammatory and antioxidant capabilities [15]. Aspirin, an anti-platelet agent, also is beneficial in the setting of FMD, where dilatations may act as a nidus for platelet aggregation. Although not a proven management strategy seen in FMD, the coincidence of aspirin use is more common in those with FMD [16]. Routine management was followed for the current patient, using first-line therapies such RAAS inhibitors and diuretics amongst others, according to the 2013 ESH/ESC Guidelines for the management of arterial hypertension [17]. However, even with intense pharmacotherapy, her blood pressure remained beyond the target range, suggesting the need for more intense therapies. 

If still not adequately managed, further pharmacologic and non-pharmacologic management options still exist. According to Acelajado et al., the standard three-drug regimen for resistant hypertension is ACEi/ARB, CCB, and thiazide diuretic [18], which is also seen in the Pathway-2 study [19]. The primary medications of the patient in this report differ from this recommendation; however, the associated comorbidities should also be considered. It has been shown that although thiazide diuretics can be more effective in non-edematous patients, loop diuretics are more effective for hypertensive patients with progressing CKD [20]. The use of CCBs also has to be carefully considered according to the patient history and pruritus and bruising after initiating nifedipine. Current considerations include an aldosterone receptor antagonist such as spironolactone. In small doses (25 mg) it has demonstrated decreases in systolic blood pressure from 14 mmHg to 36 mmHg in those with resistant types of hypertension [21]. More aggressive management options include renal denervation and potential revascularization depending on the progression of the renal artery stenosis. Renal denervation involves ablating afferent and efferent nerve supply to the renal vasculature. These nerve fibers are responsible for adjusting renal vascular resistance, renin secretion, and tubular handling of solutes including sodium to name a few [22]. Renal denervation has also been shown to reduce office blood pressure by 10 mmHg and cardiovascular events by 20% [23]; both favorable outcomes for the current patient. Further, depending on how renal artery stenosis progresses, percutaneous renal angioplasty may be considered [24]. 

The most concerning aspect of the current management of the patient in this report is their kidney function. The kidneys are responsible for both electrolyte management alongside volume status and deterioration can cause derangements of both. Further, as kidney function declines, there are fewer therapeutic options that are kidney-friendly, and monitoring overall patient status becomes more difficult [25]. Another challenge faced is the use of ACEi/ARB to treat hypertension in the case of renal artery stenosis. The function of these medications allows for efferent arteriolar vasodilation, and given the patient's history of renal artery stenosis, the combination of these two can cause a rapid decline in renal function. In a study by Chrysochou et al., 357/378 participants tolerated RAAS system inhibitors [26]. This indicates that even in those with severe renal artery stenosis, interrupting the RAAS system was beneficial and should be used unless there are absolute contraindications.

Medical management also includes lifestyle modification in concert with pharmacotherapy. Obesity is also a significant contributor to increasing blood pressure. Although the current patient is slightly overweight, this is not the main area of concern to target in this case. Rather, emphasis has been put on regular physical exercise (thrice a week) which can reduce daytime ambulatory blood pressure up to 17.8 mm Hg [27]. Further, the patient has been counseled on sodium restriction due to its direct effect on blood pressure, and according to the World Health Organization (WHO), should be limited to less than 2 grams per day [28]. The most important aspect of patient care at this time is to continuously monitor ambulatory blood pressure. 

## Conclusions

Our case underscores the complexity of managing TRH in patients with multiple comorbidities. In this case, it can be seen how the patient-specific risk factors and comorbidities were managed and the possible considerations for treatment moving forward. This includes pharmacotherapy, surgical interventions, and lifestyle modifications based on current clinical status. Continuous ambulatory blood pressure readings are constantly required to adequately monitor disease progression while lab work should be done routinely to monitor kidney function and other important parameters. Effective management requires a comprehensive approach that includes a detailed understanding of the patient’s medical history, along with pharmacological treatment and lifestyle modifications. Ongoing ambulatory blood pressure monitoring is critical to assess the patient's response to therapy, while regular follow-up visits are essential for tracking disease progression, managing comorbid conditions, and adjusting the treatment plan as needed. Given the multifactorial nature of TRH, a personalized, dynamic management strategy is paramount. Continued research into the pathophysiology and optimal treatment approaches for TRH is essential to improve outcomes for these complex patients. 
